# Accuracy of the Standard GlucoNavii Mentor in Blood Glucose Monitoring According to International Organization for Standardization 15197:2013 Criteria

**DOI:** 10.2196/20774

**Published:** 2022-05-20

**Authors:** Heeyoung Hwang, Luca Leonardi, Antonio Nicolucci

**Affiliations:** 1 SD Biosenser Suwon-si Democratic People's Republic of Korea; 2 PIKDARE S.p.A. Casnate con Bernate Italy; 3 CORESEARCH Srl Center for Outcomes Research and Clinical Epidemiology Pescara Italy

**Keywords:** blood glucose self-monitoring, diabetes mellitus, reference standards, quality control, biosensing techniques

## Abstract

This study was performed to assess the system accuracy of the blood glucose monitoring system SD GlucoNavii Mentor (SD Biosensor Inc, Korea). The study procedures were based on International Organization for Standardization 15197:2013, in that capillary blood samples from 100 participants’ fingertips were measured with three reagent system lots of the self-monitoring blood glucose system. Samples were collected for comparison measurements on a hexokinase-based glucose analyzer (Cobas Integra400 Plus, Roche Instrument Center, Switzerland). Glucose concentrations were distributed as required by International Organization for Standardization 15197. For each of the 100 evaluable samples, duplicate measurements were taken from three different reagent lots, for a total of 600 measurements. Overall, 98.3% (590/600) of individual measurement results (185/186, 99.5% for glucose values <100 mg/dl and 405/414, 97.8% for glucose values ≥100 mg/dl) were within ±15 mg/dl or ±15% of the corresponding comparison method results. All results (100%) fell into the consensus error grid zones A and B, indicating only clinically acceptable results. In conclusion, the blood glucose monitoring system SD GlucoNavii Mentor device fulfilled the system accuracy criteria of the International Organization for Standardization 15197, indicating measurement accuracy sufficient for diabetes therapy.

## Introduction

Measurement accuracy of blood glucose monitoring systems (BGMSs) is a relevant aspect in diabetes management. The International Organization for Standardization’s (ISO) 15197:2013 [1] describes requirements for BGMSs to set a minimum acceptance criteria for BGMSs’ measurement accuracy.

This study was performed to assess the system accuracy of the BGMS SD GlucoNavii Mentor (SD Biosensor Inc, Korea). According to the manufacturer, this BGMS is substantially equivalent to the BGMS Pic GlucoTest (PIKDARE S.p.A., Italy) and Pic GlucoTest Diary (PIKDARE), which has additional Bluetooth connectivity (data on file).

## Methods

The study was conducted at the Institut für Diabetes-Technologie, Forschungs- und Entwicklungsgesellschaft mbH an der Universität Ulm, Ulm, Germany. Ethics approval was obtained from the Ethik-Kommission der Landesärztekammer Baden-Württemberg (MP-2012-009). In addition, the study was exempted from approval by the regulatory authority Bundesinstitut für Arzneimittel und Medizinprodukte (95.06 - 5661 - 7848).

Study procedures were based on ISO 15197:2013, in that capillary blood samples from at least 100 participants’ fingertips were measured with three reagent system lots of the self-monitoring blood glucose (SMBG) system. Samples were collected for comparison measurements on a hexokinase-based glucose analyzer (Cobas Integra400 Plus, Roche Instrument Center, Switzerland), which is traceable according to the requirements of ISO 17511 [2]. Glucose concentrations were distributed as required by ISO 15197, and samples with concentrations ≤50 mg/dl or >400 mg/dl could be adjusted by glycolysis or glucose supplementation. A total of 114 samples from 110 participants were taken. In total, 14 samples were excluded from analysis for different reasons: the glucose concentration category was already filled, the glucose concentrations were unstable, the comparison method’s quality control measurement was out of range, and the oxygen partial pressure of adjusted samples was outside of the range found in native blood samples [3]. For each of the 100 evaluable samples, duplicate measurements were taken from three different reagent lots, for a total of 600 measurements.

For data analysis, system accuracy criteria of ISO 15197 were applied: at least 95% of each individual reagent system lot’s results have to be found within ±15 mg/dl or ±15% of comparison method results (for glucose concentrations <100 mg/dl and ≥100 mg/dl, respectively), and at least 99% of all results have to fall into clinically acceptable consensus error grid zones A and B [1,4].

## Results

Overall, 98.3% (590/600) of individual measurement results (185/186, 99.5% for glucose values <100 mg/dl and 405/414, 97.8% for glucose values ≥100 mg/dl) were within ±15 mg/dl or ±15% of the corresponding comparison method results (Table 1). All results (100%) fell into consensus error grid zones A and B, indicating only clinically acceptable results. The SMBG system exhibited small positive measurement bias, ranging from 1.7% to 3.6%.

**Table 1:** System accuracy results for the SD GlucoNavii Mentor.

Glucose concentration range	Results within prespecified ranges of the comparison method results, n (%)		
	±5 mg/dl or ±5%^a^	±10 mg/dl or ±10%^a^	±15 mg/dl or ±15%^a^
<100 mg/dl (n=186)	109 (58.6)	164 (88.2)	185 (99.5)
≥100 mg/dl (n=414)	231 (55.8)	363 (87.7)	405 (97.8)
Overall (n=600)	340 (56.7)	527 (87.8)	590 (98.3)

^a^Differences were assessed for comparison method glucose concentrations <100 mg/dl, and relative differences were assessed for comparison method glucose concentrations ≥100 mg/dl.

## Conclusions

In conclusion, in this study, the investigated BGMS SD GlucoNavii Mentor device fulfilled the system accuracy criteria of ISO 15197, indicating measurement accuracy sufficient for diabetes therapy.

## Abbreviations

BGMS: blood glucose monitoring system
ISO: International Organization for Standardization
SMBG: self-monitoring blood glucose
